# ﻿Four new and one newly recorded species of *Araeopteron* Hampson, 1893 (Erebidae, Boletobiinae, Araeopteronini), with the checklist of the genus from China

**DOI:** 10.3897/zookeys.1256.159686

**Published:** 2025-10-22

**Authors:** Yuanyuan Jin, Chunhua Yao, Huilin Han

**Affiliations:** 1 School of Forestry, Northeast Forestry University, Harbin 150040, China Northeast Forestry University Harbin China; 2 Northeast Asia Biodiversity Research Center, Northeast Forestry University, Harbin 150040, China Northeast Forestry University Harbin China; 3 Ministry of Education, Key Laboratory of Sustainable Forest Ecosystem Management, Northeast Forestry University, Harbin 150040, China Northeast Forestry University Harbin China

**Keywords:** Guizhou, new record, new species, Noctuoidea, taxonomy, Xizang, Yunnan

## Abstract

Four new species of the genus *Araeopteron* Hampson, 1893 are described: *A. maculas***sp. nov.**, *A. simaoensis***sp. nov.**, *A. submedogensis***sp. nov.**, and *A. kononenkoi***sp. nov.**, and *A. xanthopis* (Hampson, 1907) is reported for the first time from China. The new species are diagnosed based on the adult habitus and genitalia, and compared with related species. Meanwhile, the genitalia of *A. xanthopis* are illustrated and described for the first time in this study.

## ﻿Introduction

In the early studies, investigations on the genus *Araeopteron* were conducted by numerous researchers, though it was placed in different subfamilies within the family Noctuidae*sensu lato* (Hampson 1910; Inoue 1958, 1965; Nye 1975; Sugi 1982; Poole 1989; Kononenko 1990, 2003, 2005; Kononenko et al. 1998; Fibiger and Agassiz 2001; Fibiger and Hacker 2001; Fibiger and Kononenko 2008). With the establishment and stabilization of the status of Araeopteronini Fibiger, 2005, the genus *Araeopteron* was assigned to the tribe Araeopteronini within the subfamily Boletobiinae (family Erebidae). This classification has been accepted by subsequent researchers (Holloway 2011; Zahiri et al. 2012; Kononenko 2016; Han et al. 2020; Wu et al. 2020; Han and Kononenko 2021; Hirano 2021; Cheng and Han 2022).

At present, eight species of the genus *Araeopteron* have been recorded from China. The first species of this genus in China, *A. nebulosa* Inoue, 1965, was recorded from Hong Kong by Ades and Kendrick (2004). Since then, the following new species have been discovered from China: *A. dawai* Han & Kononenko, 2021, *A. medogensis* Han & Kononenko, 2021, and *A. tibeta* Han & Kononenko, 2021 from the Xizang Autonomous Region (Han and Kononenko 2021). The remaining four species: *A. amoena* Inoue, 1958, *A. canescens* (Walker, [1866] 1865), *A. fasciale* (Hampson, 1896), and *A. fragmenta* Inoue, 1965, were reported by other researchers (Fibiger and Kononenko 2008; Cao and Han 2016; Lelej 2016; Han et al. 2020; Wu et al. 2020; Cheng and Han 2022).

## ﻿Material and methods

Adults were photographed with a Nikon D700 camera before preservation. Genitalia were dissected and prepared following standard methods (Kononenko and Han 2007). Genitalia images were captured using an AOSVI Hk-830 microscope and processed with Helicon Focus v. 7.0 and Adobe Photoshop CS6. All examined specimens, including the type specimens, are deposited in the collection at Northeast Forestry University (NEFU), Harbin, China.

### ﻿Institutional (and other) abbreviations

**NEFU** Northeast Forestry University, Harbin, China

**ZMUC** Zoological Museum University Copenhagen, Denmark

**BMNH** The Natural History Museum, London, United Kingdom

**TS** Type species

**TL** Type locality

## ﻿Systematic account

**Family Erebidae Leach, [1815**]

**Subfamily Boletobiinae Guenée [1858] 1857**

### ﻿Tribe Araeopteronini

Taxon classificationAnimaliaLepidopteraErebidae

Fibiger, 2005

B0629063-C51E-5C16-95D3-1BA1B725A046

Araeopteroninae Fibiger, 2005, Esperiana 11: 25.
Araeopteronini : Holloway 2011; Zahiri et al. 2012; Kononenko and Pinratana 2013; Kononenko 2016; Wu et al. 2020.

### ﻿Genus Araeopteron

Taxon classificationAnimaliaLepidopteraErebidae

Hampson, 1893

8EA39C63-271F-5075-B2D0-9B06FF51493F

Araeopteron Hampson, 1893, Illustrations of Typical Specimens of LepidopteraHeterocera in the Collection of the British Museum 9: 33, 136. TS: Araeopteron pictale Hampson, 1893. TL: Sri Lanka.

### ﻿ Araeopteron maculas sp. nov.

Taxon classificationAnimaliaLepidopteraErebidae

0DE55EAF-89ED-5A21-AA6A-0CABEBFF10A0

https://zoobank.org/DD79D194-5E1E-461E-8013-AAB5AE29E82D

**Figures 1–6.**
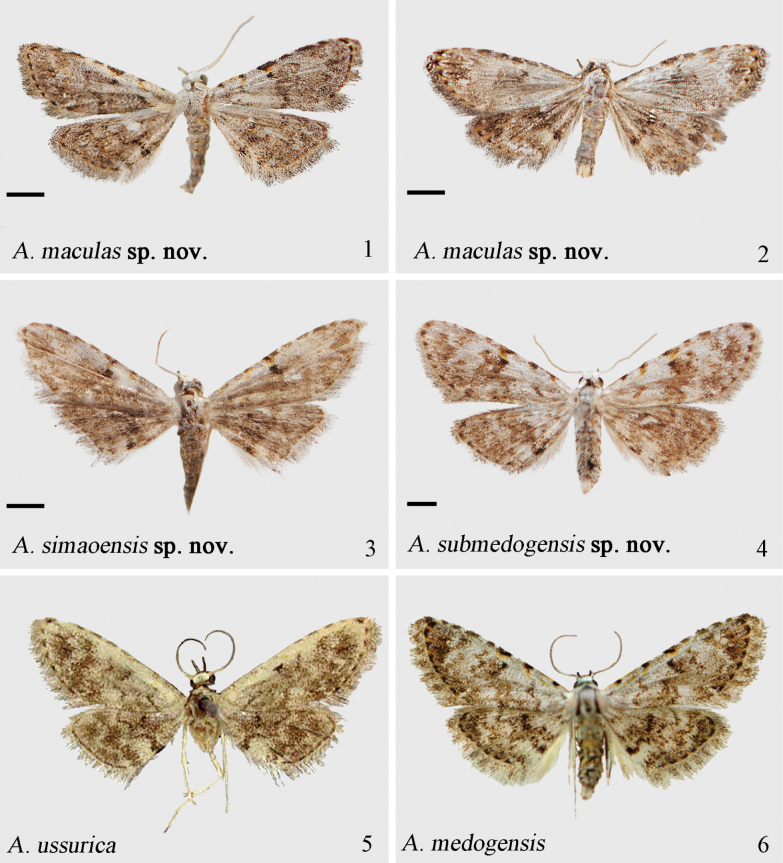
Adults of *Araeopteron* spp. 1. *A. maculas* sp. nov., male, holotype (NEFU); 2. *A. maculas* sp. nov., female, paratype (NEFU); 3. *A. simaoensis* sp. nov., male, holotype (NEFU); 4. *A. submedogensis* sp. nov., male, holotype (NEFU); 5. *A. ussurica*, male (after Fibiger and Kononenko 2008, ZMUC); 6. *A. medogensis*, male (after Han and Kononenko 2021, NEFU). Scale bars: 1 mm.

Figs 1, 2, 13, 21 Common name: 密点纤翅夜蛾

#### Material examined.

***Holotype***: China • ♂; Guizhou, Zunyi City, Xintiankan Village; 06–07.VIII.2020; HL. Han, J. Wu leg.; genit. prep. no. hhl-5011-1; in NEFU. ***Paratypes***: China • 1♂1♀; same data as holotype; HL. Han, J. Wu leg.; genit. prep. no. hhl-5013-1, hhl-5012-2; in NEFU.

#### Diagnosis.

The new species is similar to *A. ussurica* Fibiger & Kononenko, 2008 (Figs 5, 14), but it can be distinguished by external features and the structure of the male genitalia (characters for *A. ussurica* are given in parentheses):

Adult. The ground color of the forewing in *A. maculas* sp. nov. is generally whiter, with a smoky grey to grey-brown tinge (darker grayish, strongly suffused with white scales, especially at basal and costal area); the basal line is represented by two fuzzy black dots (absent); the antemedial line is obliquely inward-slanting, discontinuous, and mixed with chrome yellow (indistinct and mixed with greenish grey); the median line is wavy, more indistinct only on the costal margin, and mixed with chrome yellow (finer in the costal area, gradually becoming broader and darker towards the remaining sections, and mixed with greenish grey).

Male genitalia. The tegumen bears two distinct, triangular, paratergal sclerites (broad and curved); the saccus is approximately square in shaped (V-shaped); the uncus is short and broad (long and narrow).

#### Description.

***Adult*** (Figs 1, 2). Wingspan 9.0–10.0 mm. Antennae filiform. Head, patagia, tegula, and thorax covered with grizzled scales. Abdomen brown, interspersed with grey. Forewing grayish brown, suffused with gray, scattered with pale spots of varying sizes; antemedial and median area noticeably paler than the ground color; basal line represented by two diffuse black dots; antemedial line brown, mixed with chrome yellow, bent, obliquely inward, discontinuous; median line wavy, brown, represented by a distinct black dot on costal margin, mixed with chrome yellow; postmedial line wavy, indistinct; subterminal line blackish brown, blending with the ground color; terminal line brown, appearing as a series of dots along the veins; fringe brown, intermixed with gray; reniform stigma crescent-shaped, diffuse. Hindwing grayish brown, interspersed with grizzled scales; antemedial line indistinct; median line blackish brown, broad, curved near the Cu_2_ area; postmedial line slender, slightly wavy, suffused with white; terminal line same as on forewing; fringe slightly paler than forewing; discal spot scattered. ***Male genitalia*** (Fig. 13). Tegumen is simple at the upper half, with triangular ear-like lobe situated at basal half and having right-angled tip. Vinculum sclerotized, U-shaped. Saccus approximately square-shaped. Valva slightly sclerotized, with minute grains; costa narrow basally, gradually broadened and rounded apically; sacculus narrow, sclerotized, rounded apically, exceeding cucullus. Uncus short and broad, as long as c. 1/2 length of tegumen. Juxta weakly sclerotized. Aedeagus tubular; vesica with tiny sclerotized grains. ***Female genitalia*** (Fig. 21). Papillae anales slightly sclerotized, broad. Apophysis posterioris and apophysis anterioris slender, length of posterioris exceeding that of anterioris by 3/4 times. Antrum slightly hardened, bearing a few spurs, connecting ductus bursae and corpus bursae. Ductus bursae membranous, narrow. Corpus bursae oval-shaped.

#### Distribution.

China (Guizhou).

#### Etymology.

The species name is based on the wing characteristics of the adult moth.

### ﻿ Araeopteron simaoensis sp. nov.

Taxon classificationAnimaliaLepidopteraErebidae

3FCB897C-3C1F-5ED1-A188-1776DC886B4E

https://zoobank.org/31C7551B-DFC5-4886-ACD4-9F052908B722

Figs 3, 15 Common name: 思茅纤翅夜蛾

#### Material examined.

***Holotype***: China • ♂; Yunnan, Simao District, Manxieba Village; 22.VII.2012; HL. Han, XX. Jin, H. Geng leg.; genit. prep. no. hhl-4442-1; in NEFU. ***Paratypes***: China • 2♂; same data as holotype; HL. Han, XX. Jin, H. Geng leg.; genit. prep. no. hhl-4445-1, hhl-4452-1, in NEFU.

#### Diagnosis.

The new species is similar to *A. medogensis* Han & Kononenko, 2021 (Figs 6, 16), but can be distinguished by the following morphological characters (characters for *A. medogensis* are in parentheses):

Adult. The background color of forewing in *A. simaoensis* sp. nov. is more yellowish brown (pale grayish to whitish); the antemedial and median lines of hindwing are more distinct, black-brown between double lines (indistinct, it is lighter, grayish); the outer margin of the hindwing is slightly concave medially (sinuous).

Male genitalia. The paratergal sclerites are wider and rounder (smaller); the saccus is tongue-shaped (U-shaped); the clasper is fused to the sacculus, has a sharp and gradually narrowing harpe in 2/3 length of valva (with pointed and tapered harpe in its apical 1/3); the costa is narrow basally, gradually bulging in the apical 2/5 part of the valva (with arched bulge in apical 1/2 part of valva).

#### Description.

***Adult*** (Fig. 3). Wingspan 9.0–10.0 mm. Antennae filiform. Head, patagia, tegula, and thorax grayish white. Abdomen brown, covered with grey. Forewing pale smoky grey with brownish, adorned with black dots along the costa; basal line oblique, blurred and discontinuous, with smoky yellow; antemedial line yellow-brown, wavy, oblique; median line smoky brown, indistinct and wavy; postmedial line indistinct, rising to M_2_, then bending and continuing obliquely; median area appearing darker than rest of wing; subterminal line grayish white, indistinct and wavy; terminal line barely discernible; fringe smoky brown, mixed with white; reniform stigma round and hazy. Hindwing brown with yellowish brown, slightly darker than the forewing; antemedial line blackish brown and hazy; median line parallel to postmedial line, blackish brown, with dark brown between double lines; interspersed with tawny; fringe weakly darker than forewing; discal spot absent. ***Male genitalia*** (Fig. 15). Tegumen membranous, triangular, with broader and rounded ear lobes on both sides. Vinculum wide and thick, slightly sclerotized, V-shaped. Saccus tongue-shaped, weakly sclerotized. Valva narrowed basally; sacculus weakly sclerotized, gradually rounded apically; clasper fused to the sacculus, with a sharp, narrowing harpe, placed at c. 2/3 length from base of valva; costa narrow basally, gradually bulging apically. Uncus short, approximately as long as 1/2 length of tegumen, weakly curved, relatively sclerotized. Juxta membranous. Aedeagus short, tube-shaped, slightly curved; coecum rounded, as long as 1/4 length of aedeagus, strongly sclerotized; vesica membranous, covered with small grains. ***Female genitalia*.** Unknown.

#### Distribution.

China (Yunnan).

#### Etymology.

This species name is derived from the locality of the type, Simao.

### ﻿ Araeopteron submedogensis sp. nov.

Taxon classificationAnimaliaLepidopteraErebidae

A0C76756-1A15-51E6-AEE9-7EFCF0402E08

https://zoobank.org/75B7EBE8-4DEB-4C26-90A2-8BCB035CB8A0

Figs 4, 17 Common name: 亚墨纤翅夜蛾

#### Material examined.

***Holotype***: China • ♂; Aut. Reg. Xizang, Nyingchi City, Motuo (=Medog) County, Beibeng Township, Dergong Village; 26.V–4.VI.2021; HL. Han leg.; genit. prep. no. hhl-5016-1; in NEFU. ***Paratype***: China • 1♂; same data as holotype; HL. Han leg.; genit. prep. no. hhl-5018-1; in NEFU.

#### Diagnosis.

The new species is similar to *A. medogensis* (Figs 6, 16), but it can be distinguished by the following morphological characters (characters for *A. medogensis* are in parentheses):

Adult. The background color of forewing is pale grayish to whitish (pale grayish yellow, mixed with brown); the antemedial line formed by two brown bands, luminous bright orange at costal area, and the other part slender (continuous, brown with yellow at costal area).

Male genitalia. The paratergal sclerites are absent, almost integrated with vinculum (thin, broadened apically); the saccus is wide, U-shaped, basally thickened and sclerotized (narrow U-shaped); the clasper is absent (present with a sharp and narrowing harpe in its apical 1/3).

#### Description.

***Adult*** (Fig. 4). Wingspan 11.0–12.0 mm. Antennae filiform, with white scales. Head, patagia, and tegula covered with white; thorax grayish white. Forewing grayish white, suffused with many dark grey or blackish patches; basal line represented by a brown dot at the costal margin; antemedial line wavy, oblique, formed by two brown bands, with bright orange at costal area, its other part slender; median line, beige brown, with a black dot mixed luminous bright orange at costal area; postmedial line brown, indistinct, wavy, scattered; with dark brown between median and postmedial lines; subterminal line grayish white, wavy; terminal line brown, with a large dot on costal margin, other black and small; reniform stigma indicated by two black spots; fringe grey, mixed with brown. Hindwing grey, suffused with blackish; basal line blackish brown; transverse line blackish brown, with a black band on anal margin; marginal shade blackish brown; fringe same colour as on forewing. ***Male genitalia*** (Fig. 17). Tegumen triangular, weakly thickened. Paratergal sclerites absent. Vinculum thick, strongly sclerotized, flat, and U-shaped. Saccus wide, U-shaped, thickened and sclerotized at the base. Valva membranous, constricted at the middle; sacculus approximately 1/2 the width of valva, rounded apically; clasper absent; costa basally thin, swollen at 2/3 width of valva. Uncus short, as long as 1/2 of tegumen. Juxta membranous. Aedeagus long, narrow, slightly curved; vesica membranous, spineless. ***Female genitalia*.** Unknown.

#### Distribution.

China (Xizang).

#### Etymology.

This species name is derived from the locality of the type, Motuo (=Medog), and it is closely related to the existing *A. medogensis*.

### ﻿ Araeopteron kononenkoi sp. nov.

Taxon classificationAnimaliaLepidopteraErebidae

2E8A74C7-7B24-5239-A41D-D8AA5E6FBEE7

https://zoobank.org/D29B64B7-4135-4647-A578-761870E8F408

**Figures 7–12.**
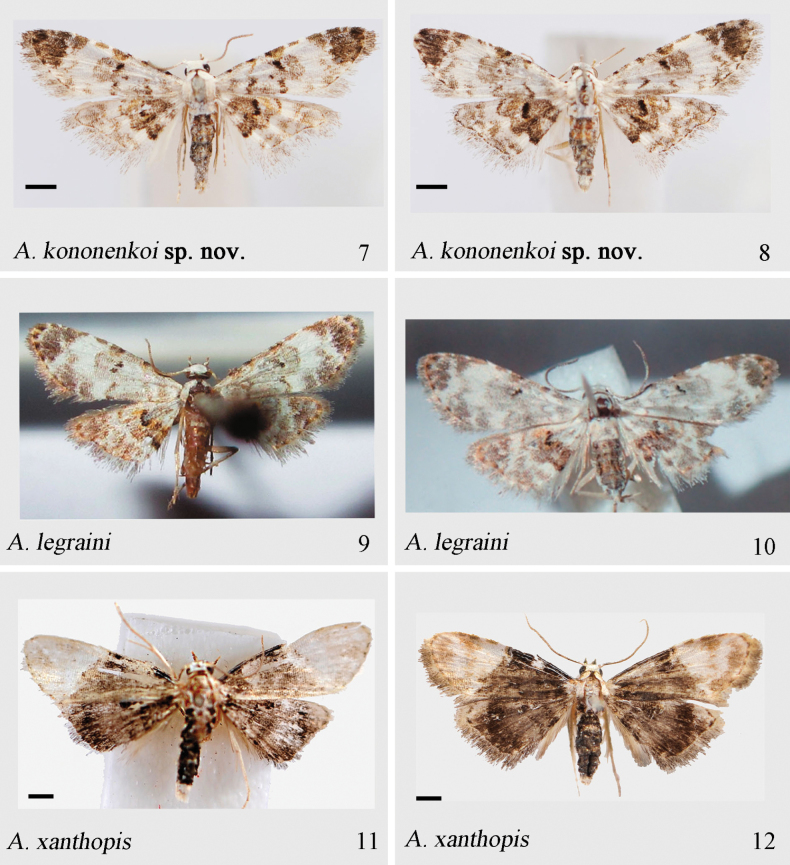
Adults of *Araeopteron* spp. 7. *A. kononenkoi* sp. nov., male, holotype (NEFU); 8. *A. kononenkoi* sp. nov., female, paratype (NEFU); 9. *A. legraini* (after Bippus 2018, BMNH); 10. *A. legraini* (after Bippus 2018, BMNH); 11. *A. xanthopis*, male (NEFU); 12. *A. xanthopis*, female (NEFU). Scale bars: 1 mm.

Figs 7, 8, 19, 22 Common name: 滇纤翅夜蛾

#### Material examined.

***Holotype***: China • ♂; Yunnan, Baoshan City, Longyang District, Baihualing Village, Mt. Gaoligong; 30.VII–2.VIII.2014; HL. Han leg.; genit. prep. no. hhl-4461-1; in NEFU. ***Paratype***. China • 1♀; Yunnan, Simao District, Beishan; 21.VII.2012; HL. Han, XX. Jin, H. Geng leg.; genit, prep. no. hhl-4460-1; in NEFU.

#### Diagnosis.

The new species is similar to *A. legraini* Bippus, 2018 (Figs 9, 10, 20, 23), but can be distinguished by external morphology and the structure of the genitalia (characters for *A. legraini* are in parentheses):

Adult. The subterminal line bears a large and brown dot at the costal margin, slender at other part (thick and flared, except delicately in the middle part); the postmedial line is reduced to a tawny bar on the inner margin (a large, irregularly scattered black patch on the inner margin); with a blackish-brown field near the apex, forming a patch at wing apex (without an additional patch at the wing apex).

Male genitalia. The tegumen is short and thin (long and thick); the cucullus is rounded, rectangular in shape (moderately broadened with a bilobed apex); the uncus is weakly sclerotized (strongly sclerotized); the vesica is membranous, without grains (covered with numerous minute grains, and with a small cornuti band).

**Figures 13–18.**
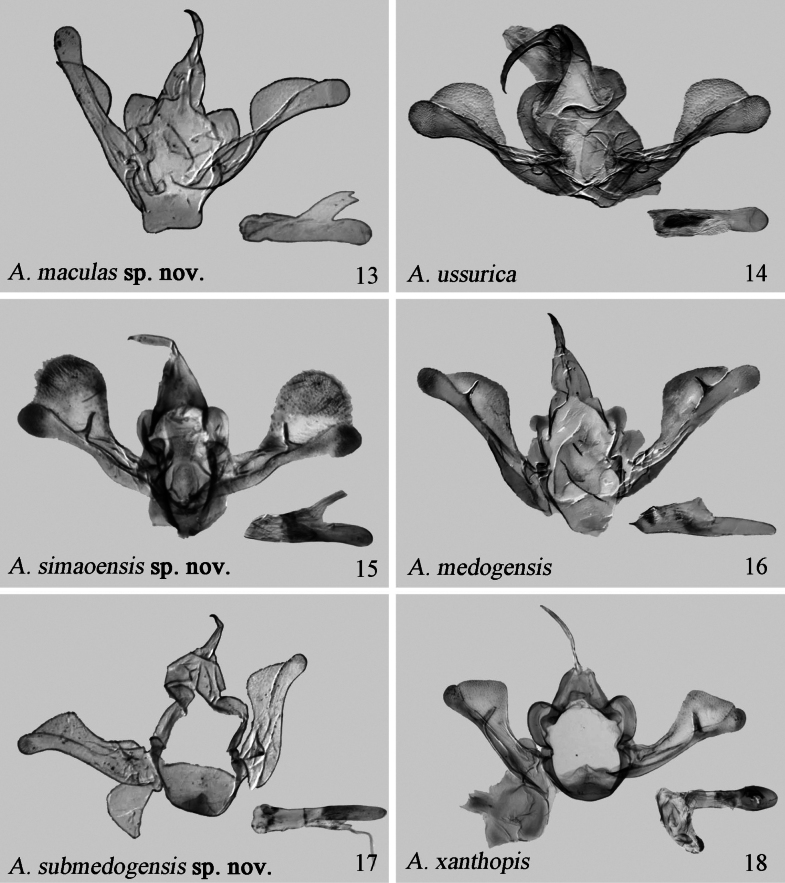
Male genitalia of *Araeopteron* spp. 13. *A. maculas* sp. nov., male, holotype (NEFU); 14. *A. ussurica* sp. nov., male, paratype (after Fibiger and Kononenko 2008, ZMUC); 15. *A. simaoensis* sp. nov., male, holotype (NEFU); 16. *A. medogensis*, male (after Han and Kononenko 2021, NEFU); 17. *A. submedogensis* sp. nov., male, holotype (NEFU); 18. *A. xanthopis*, male (NEFU).

Female genitalia. The apophysis posterioris longer than apophysis anterioris (anterioris and posterioris almost equal in length); the signum is strongly sclerotized, pie-shaped, with numerous long spines (knob-shaped, with short spines).

**Figures 19–24.**
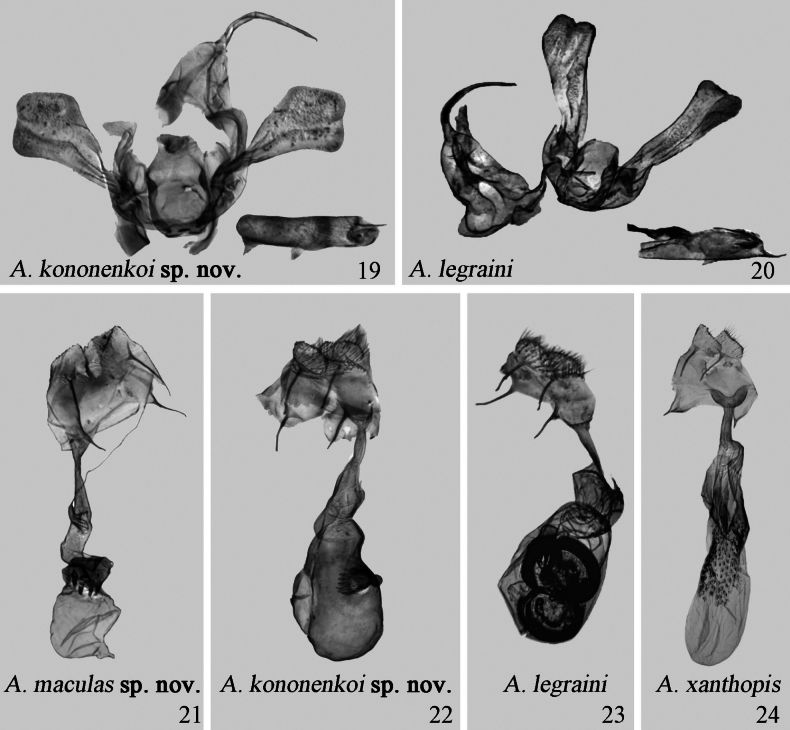
Male and female genitalia of *Araeopteron* spp. 19. *A. kononenkoi* sp. nov., male, holotype (NEFU); 20. *A. legraini*, male (after Bippus 2018, BMNH); 21. *A. maculas* sp. nov., female, paratype (NEFU); 22. *A. kononenkoi* sp. nov., female, paratype (NEFU); 23. *A. legraini*, female (after Bippus 2018, BMNH); 24. *A. xanthopis*, female (NEFU).

#### Description.

***Adult*** (Figs 7, 8). Wingspan 11.0–11.5 mm. Antennae filiform. Head, patagia, and tegula covered with cream-white scales, mixed with beige; thorax brown, covered with cream-white. Abdomen dark brownish tan, covered with off-white. Forewing cream-white, with distinct brown bands along the costal margin; basal line brown, wavy, with a black dot at costal margin; antemedial line brown, wavy, oblique, the top part showing a shade of daffodil yellow, and with a black dot on costal margin; median line, brown, diffused, only as a distinct black dot at costal margin; postmedial brown, bent in as M-shaped; subterminal line brownish yellow, double, indistinct, and wavy, with a white area between double lines and with a large and brown dot at costal margin; a blackish-brown field near the apex forming a distinct patch at wing apex; reniform stigma black; fringe brown to grayish yellow. Hindwing darker than forewing, off-white with grayish; antemedial line blackish brown; median line brown, diffused; postmedial line thin, brown, wavy and with a black dot at anal margin; median area darker than background color, blackish brown, present a white dot on 1/3 of the anal margin; subterminal line brown, double, wavy; fringe grayish white at anal margin, other part brown and mixed white; discal spot black. ***Male genitalia*** (Fig. 19). Tegumen triangular. Vinculum thick, slightly sclerotized. Saccus short, flat, arch-shaped, widened at the base. Valva narrow at the base, angled apically; sacculus slightly sclerotized, bent at its 2/3; clasper absent; costa slightly sclerotized, basally thin and narrow, gradually arched and extended to a swollen section; cucullus rounded, rectangle-shaped. Uncus long, slender, with sharp apex. Juxta large, plate-like, slightly sclerotized. Aedeagus short, slightly curved, weakly sclerotized; carinal hook slightly sclerotized, spiniform. Vesica membranous, without spines. ***Female genitalia*** (Fig. 22). Papillae anales slightly sclerotized, broad, and covered with numerous long hairs. Apophysis posterioris long and slender; apophysis anterioris short and slender, length of posterioris exceeding that of anterioris by 3/4 times. Ostium bursae funnel-shaped. Ductus bursae short, narrow basally. Corpus bursae droplet-shaped, slightly curved in the upper part. Signum strongly sclerotized, knob-shaped, and bearing numerous long spines.

#### Distribution.

China (Yunnan).

#### Etymology.

This species name is derived from honoring the researcher Dr VS Kononenko.

### ﻿ Araeopteron xanthopis

Taxon classificationAnimaliaLepidopteraErebidae

(Hampson, 1907)

2A7F8391-79DF-5180-B817-2CFF793DBD72

Figs 11, 12, 18, 24 Common name: 黑白纤翅夜蛾

Araeopterum xanthopis Hampson, 1907, Journal of the Bombay Natural History Society 17: 672. TL: Sri Lanka, Haldamulla.

#### Material examined.

China • 1♂; Yunnan, Lincang City; 6–7.IX.2014; HL. Han, MJ. Qi leg.; genit. prep. no. hhl-4431-1; in NEFU • 1♀; Yunnan, Baoshan City, Longyang District, Baihualing Village, Mt. Gaoligong; 30.VII–2.VIII.2008; HL. Han leg.; genit. prep. no. hhl-4430-2; in NEFU.

#### Description.

***Male genitalia*** (Fig. 18). Tegumen membranous, triangular. Paratergal sclerites robust and large. Vinculum thick, slightly sclerotized, U-shaped. Saccus sclerotized, mango-shaped. Valva broadened basally and apically, narrowed medially, with a bilobed apex; harpe narrow, positioned at c. 2/3 the length from valva base; costa narrow at base, gradually widening, broader than cucullus; cucullus narrow, apically rounded. Uncus slender, slightly curved. Juxta slightly sclerotized. Aedeagus tube-shaped; vesica membranous, with a single cornuti field. ***Female genitalia*** (Fig. 24). Papillae anales broad, slightly sclerotized. Apophysis posterioris and apophysis anterioris slender, sclerotized, almost equal in length. Ostium bursae U-shaped, widened, thick, and sclerotized. Ductus bursae short, narrow. Anterior part of corpus bursae slightly sclerotized, bearing small spiny grains; posterior part membranous.

#### Distribution.

China (Yunnan); Sri Lanka (Haldamulla).

#### Remarks.

Hampson (1907) first recorded this species and described the characteristics of the adult. In this study, the genitalia of this species are described and illustrated in detail for the first time.

##### ﻿Checklist of the genus *Araeopteron* Hampson, 1893 from China

***Araeopteron amoena* Inoue, 1958** 变纤翅夜蛾

*Araeopteron amoena* Inoue, 1958, Tinea 4 (1): 230, f. 2. TL: Japan, Kanagaw Prefecture, Chigasaki.

Distribution: China (Jilin, Liaoning, Shandong, Henan, Zhejiang, Hunnan); Russian Far East, South Korea, Japan.

***Araeopteron nebulosa* Inoue, 1965** 棕纤翅夜蛾

*Araeopteron nebulosa* Inoue, 1965, Tinea 7: 82, pl. 15: 4A, 4B. TL: Japan, Shizuoka Prefecture, South Izu, Odaru Spa.

Distribution: China (Jilin, Liaoning, Henan, Hong Kong); Russia, South Korea, Japan.

***Araeopteron maculas* sp. nov.** 密点纤翅夜蛾

Distribution: China (Guizhou).

***Araeopteron simaoensis* sp. nov.** 思茅纤翅夜蛾

Distribution: China (Yunnan).

***Araeopteron medogensis* Han & Kononenko, 2021** 墨脱纤翅夜蛾

*Araeopteron medogensis* Han & Kononenko, 2021, ZooKeys 1060: 23. TL: China, Xizang Autonomous Region, Motuo.

Distribution: China (Xizang Autonomous Region).

***Araeopteron submedogensis* sp. nov.** 亚墨纤翅夜蛾

Distribution: China (Xizang Autonomous Region).

***Araeopteron dawai* Han & Kononenko, 2021** 达娃纤翅夜蛾

*Araeopteron dawai* Han & Kononenko, 2021, ZooKeys 1060: 21. TL: China, Xizang Autonomous Region, Motuo.

Distribution: China (Xizang Autonomous Region).

***Araeopteron tibeta* Han & Kononenko, 2021** 藏纤翅夜蛾

*Araeopteron tibeta* Han & Kononenko, 2021, ZooKeys 1060: 25. TL: China, Xizang Autonomous Region, Motuo.

Distribution: China (Jiangxi, Xizang Autonomous Region).

***Araeopteron canescens* (Walker, [1866]1865)** 灰褐纤翅夜蛾

*Isopteryx? canescens* Walker, [1866], List Specimens lipid. Insects Colln Br. Mus. 34: 1318. TL: Queensland, Moreton Bay.

*Isopteryx? favillalis* Walker, [1866], List Specimens lipid. Insects Colln Br. Mus. 34: 1319. TL: Moreton Bay.

Distribution: China (Yunnan, Hainan); Indonesia (Borneo), New Caledonia, Australia.

***Araeopteron fragmenta* Inoue, 1965** 碎纤翅夜蛾

*Araeopteron fragmenta* Inoue, 1965, Tinea, 7: 81, pl., 15: 5A, 5B. TL: Japan, Kanagawa Pref., Fujisawa.

Distribution. China (Liaoning, Zhejiang, Hunan); Russia, South Korea, Japan.

***Araeopteron fasciale* (Hampson, 1896)** 白条纤翅夜蛾

*Araeopterum fasciale* Hampson, 1896, Fauna Br. India (Moths) 4: 543. TL: Ceylon.

Distribution: China (Hunan, Guizhou, Guangdong); Sri Lanka.

***Araeopteron kononenkoi* sp. nov.** 滇纤翅夜蛾

Distribution: China (Yunnan).

***Araeopteron xanthopis* (Hampson, 1907)** 黑白纤翅夜蛾

*Araeopteron xanthopis* Hampson, 1907, Journal of the Bombay Natural History Society 17: 672. TL: Sri Lanka, Haldamulla.

Distribution: China (Yunnan); Sri Lanka (Haldamulla).

## Supplementary Material

XML Treatment for Araeopteronini

XML Treatment for Araeopteron

XML Treatment for Araeopteron maculas

XML Treatment for Araeopteron simaoensis

XML Treatment for Araeopteron submedogensis

XML Treatment for Araeopteron kononenkoi

XML Treatment for Araeopteron xanthopis

## ﻿Additional information

### Use of AI

No use of AI was reported.

### Author ORCIDs

Yuanyuan Jin https://orcid.org/0009-0001-5724-0924

Chunhua Yao https://orcid.org/0000-0003-4076-0146

Huilin Han https://orcid.org/0000-0002-2045-6182

### Data availability

All of the data that support the findings of this study are available in the main text.
